# Draft Genome Sequence of Acidiplasma aeolicum Strain V1^T^, Isolated from a Hydrothermal Pool

**DOI:** 10.1128/mra.01046-21

**Published:** 2022-02-17

**Authors:** Aleksandr G. Bulaev, Aleksandra V. Kanygina, Andrey V. Mardanov

**Affiliations:** a Winogradsky Institute of Microbiology, Research Center of Biotechnology of the Russian Academy of Sciences, Moscow, Russia; b Federal Research and Clinical Center of Physical-Chemical Medicine of Federal Medical Biological Agency, Moscow, Russia; c Institute of Bioengineering, Research Center of Biotechnology of the Russian Academy of Sciences, Moscow, Russia; Montana State University

## Abstract

We report the draft genome sequence of Acidiplasma aeolicum strain V1^T^, isolated from a hydrothermal pool (Vulcano Island, Italy). The genome is 1.8 Mbp long with a GC content of 34%. The genome sequence was found to be closely related to those of other known strains of *Acidiplasma* genus.

## ANNOUNCEMENT

Acidiplasma aeolicum V1^T^ (DSM 18409^T^), the type of the genus Acidiplasma aeolicum, is a facultatively aerobic, moderately thermophilic, cell-wall lacked, pleomorphic archaeon oxidizing ferrous iron ions and utilizing organic nutrients that was isolated from acidic water from the hydrothermal pool (Vulcano Island, Italy) (1). It was shown that archaea of the genus *Acidiplasma* often dominate in the technological processes of biooxidation of sulfide ores and concentrates, and were also detected in a number of acidic environments in diverse geographical locations (1–6). Here, we report on the results of the assembly of the genomic sequence of Acidiplasma aeolicum V1^T^ (DSM 18409^T^).

The strain was isolated previously by Golyshina and co-authors as described in their work (1) and deposited in DSMZ-German Collection of Microorganisms and Cell Cultures (DSMZ) and Japan Collection of Microorganism (JSM) under accession numbers DSM 18409^T^ and JCM 14615^T^, respectively. To perform the present study, the strain was provided by DSMZ collection. To obtain genomic DNA, the strain was grown as recommended by DSMZ collection at 40°С in a liquid medium containing ferrous iron and yeast extract. To extract DNA, 0.5 L of the culture was grown. The cells were collected by centrifugation at 10,000 g using Allegra X22 centrifuge (Beckman, USA) and the biomass was washed twice with the acid solution containing the same component as the medium with the exception of ferrous sulfate and yeast extract. Genomic DNA was extracted using PowerSoil DNA Isolation Kit (MO BIO, USA). Genomic library was prepared using TruSeq reagents (Illumina, USA) according to the manufacturer’s recommendation. Sequencing was carried out using an Illumina HiSeq 1500 sequencer (Illumina, USA) producing 17,868,334 read pairs of 150 nucleotides (nt). Adapter sequences were removed using Cutadapt v1.8 software (7), low-quality read ends were trimmed using Sickle v1.33 software (8). In total, 358,714,350 nucleotides were used for assembly, giving a coverage of 200. The reads were assembled *de novo* using SPAdes v. 3.6.0 software (9). Default parameters were used for all software. The assembly produced 296 contigs with a total genome size of 1,778,901 bp, an N50 value of 11.5 kb, L50 of 45, and a GC content of 34.0%. Annotation of the genome sequence performed using NCBI Prokaryotic Annotation Pipeline (10) revealed 1890 coding sequences, respectively. Among these, 1,696 were identified as protein coding sequences, 46 were identified as tRNA genes, and 145 were identified as pseudogenes.

### Data availability.

Sequence data associated with the annotated genome have been deposited in the NCBI BioProject accession number PRJNA295645. This whole-genome shotgun project has been deposited at DDBJ/ENA/GenBank under the accession number LKBG00000000. The version described in this paper is version LKBG00000000.1. The Illumina reads can be found under SRA accession number SRR16381464.
